# Patients' experience of choosing an outpatient clinic in one county in Denmark: results of a patient survey

**DOI:** 10.1186/1472-6963-11-262

**Published:** 2011-10-10

**Authors:** Hans O Birk, Rikke Gut, Lars O Henriksen

**Affiliations:** 1 Region Zealand, Quality and Development, Alléen 15, 4180 Sorø, Denmark; 2 University of Copenhagen, Department of Public Health, Øster Farimagsgade 5, P.O.Box 2099, 1099 København K, Denmark; 3 Unit of Patient-Perceived Quality, The Capital Region of Denmark, Frederiksberg Hospital, Nordre Fasanvej 57, Hovedvejen indgang 13, 1. sal, 2000 Frederiksberg, Denmark

## Abstract

**Background:**

Research on patients' choice of hospital has focused on inpatients' rather than outpatients' choice of provider. We have investigated Danish outpatients' awareness and utilisation of freedom of choice of provider; which factors influence outpatients' choice of hospital, and how socio-demographic variables influence these factors in a single uptake area, where patients were free to choose any public hospital, where care was provided free at the point of delivery, and where distance to the closest hospitals were short by international standards.

**Methods:**

Retrospective questionnaire study of 4,232 outpatients referred to examination, treatment, or follow-up at one of nine somatic outpatient clinics in Roskilde County in two months of 2002, who had not been hospitalised within the latest 12 months. The patients were asked, whether they were aware of and utilised freedom of choice of hospital.

**Results:**

Fifty-four percent (2,272 patients) filled in and returned the questionnaire. Forty-one percent of respondents were aware of their right to choose, and 53% of those patients utilised their right to choose. Awareness of freedom of choice of provider was reported to be especially high in female outpatients, patients with longer education, salaried employees in the public sector, and in patients referred to surgical specialties. Female outpatients and students were especially likely to report that they utilised their right to choose the provider. Short distance was the most important reason for outpatients' choice, followed by the GP's recommendations, short waiting time, and the patient's previous experience with the hospital.

**Conclusions:**

Outpatients' awareness and utilisation of free choice of health care provider was low. Awareness of freedom of choice of provider differed significantly by specialty and patient's gender, education and employment. Female patients and students were especially likely to choose the clinic by themselves. Most outpatients chose the clinic closest to their home, the GP's recommendation and short waiting time being the second and third most important factors behind choice.

## Background

A common characteristic of public sector governance reforms in the Nordic countries in the latest two decades is a gradual development from collective systems towards an individual-based democracy model [1], where individual citizens are viewed as autonomous consumers rather than clients [2] and are expected to set priorities and allocate resources by utilising consumers' rights [3] to choose. Applied to health care, consumers - patients - may choose or be involved in the choice of:

○ Treatment

○ Individual health professional

○ Appointment time/date

○ Provider [4]

In the Nordic countries the emphasis so far is on patients choosing a provider more or less freely among several competing providers. The interest in introducing choice mostly builds on two fundamental arguments [5,6], mirroring views of choice as an end in itself or a means to an end [7]:

### The ideological viewpoint

Providing citizens with an opportunity to choose a supplier is an objective in itself, as it strengthens personal freedom [8,9].

### The instrumental viewpoint

The public sector can improve its effectiveness, reduce inequities in access to care and increase and increase its responsiveness and quality of services by introducing or strengthening *choice*, e.g. an opportunity for patients to choose a health care provider [4]. In combination with activity-based payments, where "the money follows the patient" [10-13]*choice *constitutes a self-correcting allocation mechanism, which resembles the market mechanism in competitive markets [10,14], which communicate consumers' preferences to providers more efficiently than by central planning [15], as consumers "punish" irresponsive providers by *exit *[16], leaving providers with the choice between improving the quality of their services or go out of business. Thereby individual actors' utility maximisation through rational choices, ideally, leads to an optimal resource allocation in society at the national and even international level [17,18].

The health care sector is one of the public sector areas where the introduction of *governance*-tools has been very important in countries following the Beveridge-model, like England [19], Denmark and Sweden [20], and to a lesser degree in some countries with health care organized in accordance with the Bismarck-model, like France, Germany and the Netherlands [21,22].

It is important for the effect of introduction of choice of provider on the public sector, whether consumers - e.g. patients - are aware of and utilise choice, and how they choose among different providers. A large number of studies describe inverse or negative relationships between distance to health care and its utilisation in different countries and in different institutional set-ups [23-25]. This persistent negative relationship between distance and utilisation may reflect reduced mobility in patients, judicial barriers (laws or administrative guidelines on patient referral), lack of performance data facilitating choice, and/or patients' preferences for choice of hospital.

Research into patients' choice of hospital has focused on inpatients' choices, but a large and growing share of patients are outpatients: from 2002 to 2009 the number of outpatient visits at somatic Danish hospital departments rose by 34% from 4,917,000 to 6,612,000 while the number of discharges from Danish somatic hospital departments only rose by 12% from 1,126,000 to 1,257,000 [26]. If the factors determining outpatients' choice of hospital differ significantly from those behind inpatients' choice, the assumptions underlying management and planning in the health care sector may not be valid.

On this background we investigated how outpatients chose an outpatient clinic, specifically whether awareness and utilisation differed by socio-demographic variables. Building on previous studies of Danish inpatients' choice of hospital we tested the following hypotheses, most of which were based on previous studies of Danish or Norwegian inpatients' choice of hospital:

○ Outpatients' awareness and utilisation of freedom of choice varies by specialty [27]

○ The distance between patients' home and hospitals is the most important factor behind patients' choice of hospital - most patients prefer to be treated at the hospital which is the closest to their home [23,25-27]

○ The distance to hospital is of greater importance to older than to younger outpatients, older outpatients being especially likely to choose the hospital closest to their home [28]

○ The GPs' advice strongly influences outpatients' choice of hospital [27]

○ Patients' previous experiences with a hospital strongly influences their choice of hospital; significant others' experience influence patients' choice but is of lesser importance [27]

○ Female patients are more likely than males to be aware of and utilise choice [29]

The present study was performed in Denmark where hospital care was provided free at the point of delivery by a universal, tax-financed, public health care system [30]. In the study period the citizen's home county was responsible for provision of health care performed by GPs, specialists, the county's hospitals, or other counties' hospitals (chosen by patients utilising freedom of choice or used by patients referred to hospitals performing highly specialised interventions). Each citizen had to register with a local GP, who was responsible for basic examinations and treatments. GPs, acting as gatekeepers, could refer a patient to any public hospital or a specialist for specialised services. In case of an emergency, the patient had direct access to a hospital but could not choose the hospital by themselves. GPs were self-employed and responsible for their own facilities and never performed their tasks in a hospital.

The GPs were paid by the counties in proportion to 1) the number of patients registered with them (capitation, approx. 1/3 of GPs' income), and 2) the number of services they provide to their patients (fee-for-service, approx. 2/3 of GPs' income). Specifically the payments to GPs were independent of the number of referrals or by choice of hospital. The counties and the Ministry of the Interior and Health published waiting time forecasts for common elective treatments at the hospitals on the Internet to ease patients' choice of hospital, but data on other aspects of service or clinical quality at clinics was not published systematically.

Elective patients could choose the clinic during the visit to the GP or after the visit to the GP but before going to the clinic. If one or more visits to the clinic were indicated, the patients could choose another clinic at any time before the last visit. Information to patients about freedom of choice of hospital was provided in the media, in leaflets available at general practices, and libraries and other public buildings. If patients did not make the choice by themselves, the GP chose the clinic.

If hospital personnel found indication for one or more check-ups after treatment at the hospital/outpatient clinic the patient was free to choose a specific outpatient clinic. Otherwise the hospital personnel chose the outpatient clinic.

Danish public hospitals were owned and managed by the counties. The private Danish hospital sector owned less than 1% of Danish hospital beds in the study period. Danish hospitals provide in-patient as well as outpatient care. If a patient was referred to a hospital outside the home county, the home county/region paid a DRG-charge to the county/region which owned the hospital performing the treatment, thereby creating a financial incentive to treat patients living in other counties/regions, but clinics were not obliged to accept elective patients from other counties.

## Methods

The Danish county of Roskilde, which was responsible for provision of health care in the study area until the introduction of an administrative reform by January 1 2007, performed a biannual survey of outpatients' experience with the county's outpatient clinics. In 2002 three of the 38 questions in the survey concerned outpatients' awareness and utilisation of their freedom of choice of hospital and their reported reasons for choosing the outpatient clinic.

The source group consisted of all outpatients referred to examination, treatment (including surgery), or follow-up at one or more of the 11 somatic outpatient clinics in Roskilde County in two months of 2002.

To eliminate influence of patients' experience as inpatients, patients who had been hospitalized at any Danish hospital within 12 months of attending the outpatient clinic were excluded from the study. Therefore the study included patients who attended an outpatient clinic only once or a few times, and patients who attended the clinic for a regular check-up and whose latest discharge took place more than one year before the study period.

The 11 outpatient clinics included the following specialties: Internal medicine (2 clinics), general surgery (2), orthopaedic surgery (1 clinic), rheumatology (1), neurology (1), ophthalmology (1), paediatrics (1), gynaecology and obstetrics (1), and ear, nose and throat (1). The survey of patients' experience aimed at reflecting patients' experience at all clinics, and therefore 400 patients from each outpatient clinic were randomly allocated to the study group. For clinics visited by less than 400 patients in the two months all patients were included in the study group. Patients were only included in the study group once for each outpatient clinic they attended.

Waiting time varied by specialty and by intervention and data on the intervention was not included in the questionnaire. Therefore we could not include patients' expected waiting time in the study.

The study group received a standardised questionnaire developed for use in a biannual nationwide survey of inpatients' experience with Danish public hospitals. The original version of the questionnaire was validated for readability and understanding by interviews with 80 inpatients and was used for two nationwide studies of Danish inpatients' experience. Unit of Patient Evaluation, Copenhagen County, Denmark, (UPECC, now renamed "the Unit of Patient-Perceived Quality, Capital Region of Denmark") revised this questionnaire for use by outpatients and validated the questionnaire by interviews with 12 patients from five outpatient clinics using an interview guide developed by UPECC. See additional file 1: Extract from the questionnaire.

Compared to the standardised questionnaire the language was adjusted to the outpatient-clinic setting, we referred to the media rather than the clinic's "reputation", and we added family and friends' experiences as potential reasons for choosing the clinic.

Socio-demographic data on each patient in the study group included:

○ Specialty

○ Gender

○ Age

○ Education

○ Employment

The questionnaire did not include questions concerning patients' use of published data on clinics' quality or service level.

The study was performed anonymously. All members of the study group received one reminder by mail.

Data were entered into a database (SAS).

We weighted the responses from each specialty in accordance with the specialty's share of the number of outpatients which attended the clinics during the study period and met the inclusion criteria.

Respondents' and non-respondents' specialty, gender, and age were compared by a univariate chi^2^-test.

Respondents' awareness and utilisation of free choice of hospital was analysed by gender, education, and employment by a univariate chi-square test.

Respondents aware of and utilising free choice of hospital were compared by gender, age (0-60 vs. 61+ years), referring doctor, education (none/short, medium and long), and specialty category (surgical vs. medical specialty but *not *by single specialties), using a logistic regression analysis, which did not control for other factors. Level of significance: 5%. "Surgical specialties" included general surgery, orthopaedic surgery, ophthalmology, gynaecology and obstetrics, and ear, nose and throat, while "medical specialties" included internal medicine, neurology, and paediatrics.

Respondents' reasons for choice of hospital were analysed by specialty category (surgical vs. medical specialties but not by single specialty), gender, education (none/short, medium and long), employment (in employment vs. other), and age (0-60 vs. 61+ years) using a univariate chi-^2^-test.

The study was performed in accordance with the Helsinki Declaration. According to section eight in the Danish Act on a Biomedical Ethics Committee System and the Processing of Biomedical Research Projects questionnaire studies like the present study are not notifiable to the Danish research ethics committee system, if the study does not include biological material [31].

## Results

### Respondents and representativeness

The study group included 4,232 patients; 2,272 (54%) filled in and returned the questionnaire. The respondents did not differ significantly from the study group but due to the recruitment method the unweighted study group differed from a random sample of outpatients. Female patients (response rate: 56%), patients attending a clinic of gynaecology/obstetrics (59%) and patients aged 40-79 years (58%) were especially likely to respond. Male patients (50%), patients aged 0-29 years (41%), and patients attending a clinic of neurology (46%) were the least likely to respond.

On average the respondents had attended the clinic four times within the latest 12 months. When the respondents filled in the questionnaire 20% had visited the clinic only once, and 41% had visited the clinic two or three times, within the latest 12 months.

### Patients' reported awareness of freedom of choice (weighted respondents)

Forty-one percent of the respondents reported that they were aware of their right to choose the hospital (Table 1). Patients' reported awareness differed significantly by specialty, patients referred to clinics of ophthalmology, ear, nose and throat, gynaecology/obstetrics and orthopaedics (the surgical specialties) being especially likely to report that they were aware of their right to choose. Female patients, patients with longer education and salaried employees in the public sector were significantly more likely to be aware of their right to choose than other patient groups.

**Table 1:** Weighted respondents' characteristics and reported awareness of and utilisation of freedom of choice of hospital.

Patients characteristics		Response rate (%)	Respondents' reported awareness	Reported use of choice
Gender	Men	58	38*	52**
	Women	42	43*	63**
Age (years)	0-9	5	41	53
	10-19	5	31	40
	20-29	4	33	71
	30-39	10	44	65
	40-49	13	42	58
	50-59	22	45	56
	60-69	20	42	56
	70-79	14	39	63
	80+	6	38	66
Specialty	Rheumatology	9	34**	63
	Internal medicine	27	39**	58
	Surgery	17	39**	56
	Neurology	5	34**	46
	Ophthalmology	6	54**	70
	Ear, nose and throat	12	46**	57
	Paediatrics	5	33**	36
	Gynaecology/- obstetrics	7	46**	57
	Orthopaedics	13	45**	62
	Surgical specialties	55	44**	60
	Medical specialties	45	37**	56
Referring doctor	GP	51	40	62
	Specialist	27	43	55
	Ambulatory	7	40	53
	Other	14	40	54
	Does not remember	1	22	75
Education	Very short	32	37*	57
	Short	40	44*	56
	Medium or long	29	46*	63
Employment	Student	3	35*	78**
	Non-skilled labor	4	42*	64**
	Skilled labor	6	39*	61**
	Salaried employees, priv.	17	37*	59**
	Salaried employees, publ.	22	49*	58**
	Self-employed	8	44*	63**
	Unemployed	13	39*	38**
	Pensioners	26	40*	66**

**: p < 0.01 *:p < 0.05

In logistic regression analysis involving gender, age and education female patients and patients with longer education were significantly more likely to report that they were aware of their freedom of choice, like in univariate analysis (Table 2).

**Table 2:** Weighted respondents' reported awareness/utilisation of freedom of choice of hospital, logistic regression, adjusted odds ratios.

Factor		Unit	Odds ratio	**Lower 95%-conf**.	**Upper 95%-conf**.
Awareness	Female vs. male patients	1.00	1.234*	1.001	1.522
	Age: 0-60 vs. 61+ years	1.00	0.960	0.768	1.201
	Short/no education vs. long	1.00	0.695**	0.532	0.907
	Medium education vs. long	1.00	0.902	0.704	1.156
Utilisation	Female vs. male patients	1.00	1.462*	1.043	2.049
	Age: 0-60 vs. 61+ years	1.00	1.033	0.720	1.482
	Short/no education vs. long	1.00	0.801	0.519	1.237
	Medium education vs. long	1.00	0.835	0.566	1.234

*: p < 0.05: **: p < 0.01

### Utilisation of choice among patients aware of freedom of choice (weighted respondents)

Fifty-three percent of respondents, who reported that they were aware of their right to choose, reported that they utilised this right.

In univariate and logistic regression analysis female patients were significantly more likely than men to report that they chose the hospital (Table 1, Table 2).

The share of parents which utilised free choice of hospital on behalf of their children was markedly lower than in other patient groups, even though the share of parents who were aware of free choice was lower than in other patient groups. Reported utilisation of free choice was also low in patients referred to outpatient clinics in neurology, while utilisation was high in patients referred to ophthalmology (where awareness also was high). Reported utilisation was no higher in patients referred to surgery than in patients referred to internal medicine.

Patients who had an education of long duration, and patients who were self-employed or salaried employees in the public sector were especially likely to choose the hospital (Table 1). The statistically significant univariate association between education and utilisation of choice disappeared in logistic regression (Table 2), unlike the association with awareness of choice.

Patients who were 20-39 years old were also especially likely to choose the hospital, but age was not a statistically significant variable (Table 1, Table 2).

### Reasons for choice of hospital

Distance to hospital was the factor which the greatest number of patients reported to be important for their choice (Table 3), followed by the GP's recommendation and the waiting time's length.

**Table 3:** Weighted respondents' reported reasons for choice of hospital by specialty category, gender, education, employment and age.

Respondents' characteristics		Short distance	GP's recommendation	Waiting time	Patient's experience	Other reasons	Friends' experience	Family's experience	Media reports
Specialty	Surgical	40	23	30****	21	11	10*	7	2
	Medical	50	27	14****	22	16	4*	4	4
Gender	Female	47*	26	19**	25*	15	8	6	2
	Male	37*	22	33**	16*	10	5	6	3
Education	None/very short	35	25	23	23	10*	9	7	1
	Short	47	24	22	17	12*	7	5	1
	Medium/long	42	24	25	24	21*	8	5	5
Employment	In employment	43	25	23	17*	16	5	5	2
	Other	43	23	26	27*	11	9	6	4
Age (years)	0-60	44	25	22	19	17**	6	5	3
	61+	43	22	28	27	7**	9	7	2
Total		44	24	24	22	13	7	5	3

*: p < 0.05; **: p < 0.01; ****: p < 0.001

Respondents could state more than one reason for choice.

Twenty-two percent of outpatients reported that their own experience influenced their choice of hospital, while seven percent were influenced by their friends' experience, and five percent by their family's experience. Media reports were only referred to by three percent of the patients.

Female patients were significantly more likely than men to choose the clinic closest to their home, while male patients and patients referred to surgical specialties were significantly more likely to make their choice based on waiting time than female patients and patients referred to medical specialties. Male patients and patients out of employment (including pensioners) were significantly more likely to make their choice based on their personal experience with clinics than female patients and patients in employment. Friends' experience was especially important to patients referred to surgical specialties, and patients with longer education and younger patients were especially likely to refer to reasons which were not listed in the questionnaire.

## Discussion

### Awareness of freedom of choice

Questionnaire studies of Danish inpatients' awareness and utilisation of choice found a higher overall awareness of freedom of choice (more than 80%) than the present study of outpatients [27,32-34], even though the study was performed within the same health care system. The results regarding differences between specialties almost resembled results from a study of inpatients performed in the same county: inpatients referred to departments of internal medicine, ophthalmology, or ear, nose and throat were especially likely to be aware of choice, while awareness was the lowest in patients referred to geriatric departments and in parents of children referred to a paediatric department [33].

### Utilisation of freedom of choice

Patients' utilisation of choice varied by specialty. Two reasons for paediatric patients' low utilisation of free choice could be a low number of paediatric departments and quite long distances between these departments, and parents may have to pick up their child at a kindergarten or school before they can go to the clinic, leading to an even longer transport distance for the parents, making them especially sensitive to the distance to the closest clinic. In the present study we did not disaggregate from specialty level to diagnoses or the severity of the specific disease. Concern about severe diseases may make patients more likely to utilise freedom of choice of hospital, and in general in patients are more likely than outpatients to suffer from serious diseases [4].

In univariate and logistic regression analysis female patients were more likely than men to report that they chose the hospital. Likewise Norwegian female inpatients are more likely to utilise choice than male inpatients [29]. The present study provides no explanation for this difference, but women are hospitalized more often than men due to childbirth, and a longer average lifespan etc. and this greater experience may facilitate utilisation of choice.

Although age was not a statistically significant factor behind reported utilisation of choice in the present study other studies indicate that younger patients may need assistance to think through what is important to them [35], and despite older patients' reduced mobility their greater experience with providers and choice of provider may make them active choosers, although accessing and utilising data on the internet may constitute a challenge [35].

US studies have found that patients' travel distances grow with their educational level, indicating a positive association between education and mobility: patients with higher education on average earn higher incomes and may be more likely to own a car, and are more likely to live in urbanized areas with access to public transport [4]. In a French interview study pregnant women with a higher educational level were especially likely to choose a maternity unit with special technical attributes [36].

We found no studies of an association between employment and choice.

The Danish population is quite homogeneous with regard to socio-demographic variables, which may make it difficult to show statistically significant differences in behaviour between social groups.

### Reasons for choice of hospital

Short distance was the most important factor behind choice of hospital in similar Danish studies [27,32-34] and the present study where female patients were significantly more likely than men to make their choice based on short distance. Many other studies have, by use of different methodologies, found that the distance to alternative hospitals is very important for patients' choice of hospital [37], one US registry study finding that equal shares of patients chose the hospital closest to their home in rural and metropolitan areas, suggesting that there is no lower threshold below which short distance loses importance [38]. Distance interacts with other patient- and disease-specific factors like patients' age [28], but in the present study gender was the only statistically significant factor behind choice. Institutional differences between health care services in the US and in a Beveridge-system like Danish health care complicate international comparisons.

The GP's recommendation and waiting time were the second most important factor behind patients' choice of clinic.

In 2002 20% of Danish inpatients who chose a specific hospital reported that the GP influenced their choice [32]. The present study indicates that the GP plays an even larger role for outpatients' choice of provider than for inpatients.

Danish studies have consistently found that (short) expected waiting time is the fifth most important factor behind inpatients' choice [27,32-34]. The great importance of (short) waiting time to patients referred to clinics of surgical specialties is not surprising as the share of elective patients is higher in surgical specialties than in medical specialties. Studies of patients' acceptance of waiting time indicate that its numerical length is very important to patients: cataract patients generally accept waiting times of three months and less, while waiting times of six months or more are likely to be perceived as too long [39,40]. In British studies of patients on a waiting list, with very long and uncertain waiting times, who were offered early treatment at a distant hospital, all or a major share of the patients were willing to travel far to reduce their waiting time [41-44]. It was not possible to distinguish between the effect of waiting time itself and uncertainty about its length. In a hypothetical study patients reported that for every additional hour of travel they would, on average, require a reduction in waiting time of 2.3 months to take up the offer of treatment at a distant hospital. A choice of a hospital abroad required a reduction in waiting time of around 5.9 months [44]. Cataract patients' perception of waiting times for surgery being too long is not associated with their demographic characteristics [40]. A study of patients waiting for hip or knee implantation found a moderate inverse association between on the one hand severity categories like bodily pain and physical functioning, and acceptable waiting time on the other [45].

The present study does not provide information on whether the patients who point to their own previous experience with the hospital have been treated at the same department or at another department. A study from the UK found that a previous negative experience with a local hospital was the strongest predictor for willingness to choose a non-local hospital [4].

Family and friends' experience played a minor role for choice - probably partly because the hospital was chosen during a visit to the GP where it is difficult to approach other people and ask them about their experience, before the visit is over, although patients were free to call the GP's secretary and ask for a re-referral if they changed their mind after the visit to the GP. Studies of clinically healthy citizens' hypothetical choice of hospital indicate that patients' experience and other people's reports of their experience with certain hospitals is more important or just as important for the choice of hospital as the GP's advice and published information about hospitals' quality [46-48].

Positive media reports about the clinic played a minor role for the patients' choice. The patients may find it difficult to remember media reports about each and every hospital they are able to choose among. According to a US study reports of single sensational events at a hospital is more important for patients' choice of a hospital than data on general mortality [49].

### Implications

A major reason for the introduction of proxy markets, like free choice of hospital in combination with activity-based financing is the assumption that this system will push hospital managers to improve quality and service. This management concept depends on that patients are aware of and willing to choose the hospital based on quality and service. The present study indicates, that outpatients' choice behaviour will send relatively weak signals to hospitals compared to inpatients, because relatively few outpatients are aware of and utilise free choice of hospital, and because a large share of the outpatients choose the hospital based on which hospital is the closest, which is independent of hospitals' quality or service.

Waiting time influences patients' choice, but the relatively small share of patients who choose the hospital based on waiting time data will not be sufficient to level out waiting times at different hospitals.

The study was performed at a time when only little information was published about hospitals' quality and service levels. In such situations proxy-measures of service and quality like the GP's, the patient's, and family and friends' experience constitute important factors behind choice, which means that outdated data on quality and service may play a major role for patients' choice.

Assumptions on outpatients' preferences for choice of provider should build on studies of outpatients rather than generalizations from studies of inpatients' preferences and choices.

Further research into outpatients' choice behaviour and utilisation of data sources is warranted and should distinguish between referrals of outpatients to a clinic and outpatients attending a clinic for a check-up for an ongoing medical condition or after hospitalisation.

### Limitations of the study

A medium response rate increased the risk of selection bias, because some patient groups may be especially likely to answer, e.g. patients with strong views on choice of hospital probably were more likely to participate in the present study than other patients, and female patients, who on average were more likely to be aware of and utilise choice than male respondents, were also more likely to respond. Therefore the present study may exaggerate 1) the importance of any single factor for choice, 2) patients' likelihood to choose the hospital by themselves, and 3) factors which are especially important to female patients.

The present study includes many statistical tests and some of the statistically significant findings in univariate analysis may be due to mass significance rather than causality.

The study group received the questionnaire approximately three months after attending the outpatient clinic. Recall bias may constitute a problem. This is especially so because chronic patients attending a regular check-up may have chosen the clinic several years before the present study was performed. Furthermore respondents may have provided a simplified description of the decision process, thereby exaggerating the influence of the most important reasons for their choice.

The respondents' participation in the study may have led them to describe a decision making process which is more rational than their real choice behaviour - for example by exaggerating their awareness and utilisation of choice, and the influence of supposedly rational reasons for choice like short waiting time and the GP's advice, while underreporting reasons which may be considered to be less rational, like short distance to hospital and informal information sources, like family and friends.

However, most of the questions in the questionnaire concerned their experience ('patient satisfaction'); only three of 38 questions concerned the patients' choice of outpatient clinic, and therefore we assume that the subjects' participation in the study may only have had little impact on their responses.

The study did not provide evidence on outpatients' utilisation of published data on quality or service in choice of provider.

The study was performed nine years after the introduction of freedom of choice of (public) hospital in Denmark and before freedom of choice was extended to include some private clinics. Danish patients have become increasingly used to utilising freedom of choice. Therefore the results from the present study may be more representative of patients' behaviour in public health care systems (Beveridge- or NHS-health care systems) several years after the introduction of choice than immediately after its introduction. The results and conclusions should only be generalized to other institutional settings with caution.

## Conclusions

Nine years after the introduction of free choice of public hospital/outpatient clinic in Denmark, outpatients' awareness and utilisation of free choice of health care provider was low. Awareness of freedom of choice of provider differed significantly by specialty and patient's gender, education and employment. Female patients and students were especially likely to choose the clinic by themselves. Most outpatients chose the clinic closest to their home, the GP's recommendation and short waiting time being the second and third most important factors behind choice.

## Competing interests

The authors declare that they have no competing interests.

## Authors' contributions

All authors conceived and designed the study; RG developed the questionnaire, collected the data, and assisted in performing the statistical analyses and in writing the manuscript. HOB analysed the data and wrote the manuscript. LOH assisted in writing the manuscript. All authors read and approved the final manuscript.

## Pre-publication history

The pre-publication history for this paper can be accessed here:

http://www.biomedcentral.com/1472-6963/11/262/prepub

## Supplementary Material

Additional file 1 **Extract from the questionnaire**. The seven questions concerning outpatients' choice of hospital in the questionnaire used for investigation of patients' experience with outpatient clinics. Click here for file
