# Supplementary material for: Patients' experience of choosing an outpatient clinic in one county in Denmark: results of a patient survey
Source: BMC Health Serv Res. 2011 Oct 10;11:262. doi: 10.1186/1472-6963-11-262 (PMC3205034; doi:10.1186/1472-6963-11-262)
Supplement: Additional file 1 — Extract from the questionnaire. The seven questions concerning outpatients' choice of hospital in the questionnaire used for investigation of patients' experience with outpatient clinics. [file 1472-6963-11-262-S1.DOC]

**Additional file 1: Extract from the questionnaire:**

**Question 3: Who referred you for examination or treatment at the out-patient clinic?**

My own GP □

A specialist □

An emergency ward □

Another out-patient clinic □

I do not remember □

Other □

**Question 5: Did you know that that you could choose by yourself which outpatient clinic you were to be examined or treated in?**

Yes □

No (go to question 9) □

**Question 6: Did you choose by yourself which outpatient clinic you were to be examined or treated in?**

Yes □

No □

If you chose the out-patient clinic by yourself, we would like to know, why you chose this specific out-patient clinic.

**Question 7: Why did you choose to be examined or treated at this outpatient clinic?**

(you may tick off more than one reasons)

The clinic was the closest one to my home. □

The clinic offered the shortest waiting time [among clinics]. □

Because of positive coverage in the papers, in TV or on the radio. □

My GP recommended the clinic. □

I had had positive experiences with the clinic. □

My family had had positive experiences with the clinic. □

My friends had had positive experiences with the clinic. □

For other reasons ____________________________________________________________ □

**Question 35: What is your gender?**

Female □

Male □

**Question 36: In which year were you born?**

Year: ______

If the patient is a child, please report education (question 37) and employment status (question 38) for the parent who fills in the questionnaire on behalf of the child.

**Question 37: What is your education?**

(If you have completed more than one of the educations mentioned below, please tick off the one you completed most recently)

Basic school (9 years or less) □

Basic school (10 years) [and two older educations of similar length] □

Short education (for example nursing aide, bus driver, refuse collector) □

Vocational training (for example clerk, workman, farmer) □

Upper-secondary school level □

Short higher education (1-2½ years) (for example language secretary, computer specialist) □

Medium long higher education (3-4 years) (for example nurse, teacher, BS or BA) □

Long higher education (more than 4 years) (for example engineer, MD) □
